# The molecular basis for ethnic variation and histological subtype differences in prostate cancer

**DOI:** 10.1007/s11427-013-4522-0

**Published:** 2013-07-13

**Authors:** Yang ZONG, Andrew S. GOLDSTEIN, JiaoTi HUANG

**Affiliations:** 1 Howard Hughes Medical Institute, University of California, Los Angeles, CA 90095, USA; 2 Departments of Molecular and Medical Pharmacology, University of California, Los Angeles, CA 90095, USA; 3 Pathology and Laboratory Medicine, University of California, Los Angeles, CA 90095, USA; 4 Urology, University of California, Los Angeles, CA 90095, USA; 5 Jonsson Comprehensive Cancer Center, David Geffen School of Medicine, University of California, Los Angeles, CA 90095, USA; 6 Eli and Edythe Broad Center of Regenerative Medicine and Stem Cell Research, University of California, Los Angeles, CA 90095, USA

**Keywords:** prostate cancer, castration resistance, neuroendocrine differentiation, small cell neuroendocrine carcinoma

## Abstract

Prostate cancer is a common malignancy among men in Western countries. Recently the morbidity and mortality of prostate cancer increase dramatically in several oriental countries including China. Rapidly evolving technology in molecular biology such as high-throughput sequencing and integrative analysis of genomic and transcriptomic landscapes have enabled the identification of key oncogenic events for prostate cancer initiation, progression and resistance to hormonal therapy. These surging data of prostate cancer genome also provide insights on ethnic variation and the differences in histological subtype of this disease. In this review, differences in the incidence of prostate cancer and the prevalence of main genetic alterations between Asian and Western populations are discussed. We also review the recent findings on the mechanisms underlying neuroendocrine differentiation of prostate cancer and the development of small cell neuroendocrine carcinoma after androgen deprivation therapy.

Prostate cancer is the second most commonly diagnosed nondermatologic cancer and the sixth leading cause of cancer-related deaths in men worldwide [1]. The morbidity and mortality of this hormone-related cancer vary significantly among different countries and racial/ethnic populations. In western countries, prostate cancer is the most prevalent malignancy for men [1]. In 2013, approximately 238590 new cases of prostate cancer are expected in the United States, which account for 28% of all newly diagnosed cancers in American men, and it is estimated that about 29720 American men will die from this disease this year [2]. In several developing countries located primarily in the Caribbean, South America and sub-Saharan Africa, prostate cancer surpasses lung cancer and gastroenterological cancer and is the leading cause of cancer deaths in men [3].

## 1 The difference in the incidence of prostate cancer among different ethnic groups

In contrast, the incidence and mortality rates of prostate cancer are much lower in Asian countries including China, India, Japan, Philippines, South Korea, Thailand and Vietnam [3,4], although it has increased significantly in most of these countries in recent years, with about 12%–14% average annual increase in China and South Korea [3]. The significant differences in the incidence of prostate cancer between Asian countries and Western countries may be attributed to dietary and lifestyle-related factors, as well as differences in the use of prostate specific antigen (PSA) screening and access to medical care [4]. However, the epidemiological studies of Asian immigrants living in North America or European continents reported that the incidence and mortality rates of prostate cancer among these Asian immigrants are 50%–80% lower than those for non-Hispanic whites and Africa Americans [2,4], suggesting that in addition to environmental influences, genetic heterogeneity also contributes to prostate carcinogenesis.

To identify genetic variants associated with prostate cancer risk, multiple genome-wide association studies (GWAS) have been carried out in populations of European descent, African-Americans, Japanese and Chinese. So far 77 single nucleotide polymorphisms (SNPs) associated with prostate cancer susceptibility have been identified [5,6], including two new risk loci, 9q31.2 and 19q13.4, which were found to be significantly associated with predisposition to prostate cancer in a Han Chinese population [7], and 23 new susceptibility loci that were recently identified using the iCOGS custom genotyping array in a linkage study with a larger pool of samples of European ancestry [5]. Unlike the previously identified loci that were associated exclusively with non-aggressive prostate cancer, 16 out of these 23 new susceptibility loci were found to be associated with aggressive as well as non-aggressive disease, although none of the new loci are associated exclusively with the indolent form of prostate cancer [5].

Of the 23 newly identified susceptibility loci for prostate cancer, rs11650494 is located at chromosome 17q21, a gene-dense locus that contains several prostate cancer candidate genes such as *HOXB13* and *SPOP* [5]. Interestingly, by exome sequencing 202 genes on chromosome 17q21–22, a rare but recurrent germline mutation in the *HOXB13* gene (HOXB13 G84E) was reported to be highly associated with familial prostate cancer in Caucasians [8]. Although the same mutation was not detected in Chinese men, a novel rare mutation (G135E) in *HOXB13* was found to be associated with increased prostate cancer risk among Chinese men [9]. HOXB13 is a member of the homeodomain family of transcription factors, which has been implicated in normal prostate development [10] as well as cancer pathogenesis in tumors of several epithelial tissue origins [11–13]. However, the definitive role of HOXB13 mutations in prostate carcinogenesis remains unclear. Similarly, Barbieri et al. [14] described that 6%–13% of localized primary prostate cancer samples harbor mutations in *SPOP*, a gene encoding the substrate-binding subunit of a cullin-based E3 ubiquitin ligase. While wild-type SPOP protein interacts directly with steroid receptor coactivator SRC-3 and enhances its ubiquitin-dependent proteasomal degradation to control the transcriptional activity of androgen receptor (AR), it has been recently demonstrated that most prostate cancer-associated SPOP mutants lack the capacity to promote the turnover of SRC-3 protein and thus display attenuated tumor suppressor effects in prostate cancer cell lines [15]. Consistently, *in vitro* studies showed that forced expression of SPOP mutant or siRNA-mediated knockdown of SPOP led to increased invasion, but no significant changes in cell growth and viability [14], suggesting that SPOP protein could function as a tumor suppressor in the prostate.

## 2 Differences in the prevalence of prostate oncogenic events among different ethnic groups

In the past decade, rapidly evolving technologies have revolutionized our understanding of the cellular and molecular basis for the development of prostate cancer. In addition to the aforementioned SNPs associated with prostate cancer susceptibility, a variety of genetic and epigenetic alterations have been found to be involved in prostate cancer initiation, progression, metastasis and drug resistance. Recurrent gene fusions involving several members of ETS transcription factor family (*ERG, ETV1, ETV4* or *ETV5*) were found to be the most frequent genetic alterations in prostate cancer, which can be detected in as many as 50%–70% of prostate cancer samples [16,17]. *ETS* gene fusions resulting from either interstitial deletion or chromosomal translocation lead to the generation of a handful of fusion transcripts that commonly contain 5′ regulatory elements from androgen-responsive genes, such as *TMPRSS2*, and the coding sequence of ETS transcription factors. The *TMPRSS2-ERG* fusion at chromosome 21q22 is the predominant subtype of *ETS* fusions [17,18]. The prevalence of the *TMPRSS2-ERG* fusion in prostate cancer appears to vary in different ethnic groups, with the highest frequencies of occurrence in Caucasians (~50%) [19,20], modest in African Americans (24%–31%) [19,20] and much lower frequencies in Asian populations (8%–21%) [19,21–23], indicating that distinct genetic alterations may drive prostate cancer development in different ethnic groups (Table 1).

Similar ethnic differences have also been demonstrated in PTEN status, another common early event involved in prostate carcinogenesis. In Western countries, loss or alteration of at least one *PTEN* allele is frequently present in primary prostate cancer, and is correlated with disease progression to the metastatic stage [31,32]. It has been shown that approximately 40%–70% of primary prostate tumors have PTEN deletion, resulting in activation of the PI3K-AKT pathway [30,31]. By parallel comparison of the frequencies of PTEN deletion/inactivation among prostate cancer samples from China and the United Kingdom, Mao et al. [24] revealed that only 34% of Chinese tissue specimens displayed low levels of PTEN, although the frequency of PTEN inactivation (69.8%) in specimens from the United Kingdom were similar to that previously published for Western samples.

The low prevalence of *ERG* gene fusion and PTEN deficiency in Chinese patients with prostate cancer suggests that alternative molecular mechanisms may play important roles in the development of prostate cancer in Asian men. Using RNA-seq technology to profile the changes in the transcriptome of primary prostate cancer samples from China, Ren et al. [21] reported that two novel gene fusions, *CTAGE5-KHDRBS3* and *USP9Y-TTTY15*, occurred at high frequencies (~35%) in Chinese patients [21]. Two additional gene fusions, *SDK1-AMACR* and *RAD50-PDLIM4* were also found with relatively lower prevalence (24%–28%) in this cohort of Chinese descent. Although the functional relevance and clinical significance of these novel gene fusions remain elusive, the high recurrence of these gene rearrangements indicates that unique genetic alterations in alternative pathways may affect prostate oncogenesis among Asian patients.

In addition, the differences in activated RAS-RAF-MAPK signaling pathway also have been characterized between Asian and Western patients with prostate cancer. Although constitutive activation of RAS-RAF-MAPK pathway occurs in a majority of advanced prostate tumors, the incidence of direct mutations of the upstream activators such as KRAS and BRAF are not commonly found in prostate cancer [31]. However, it has been reported that the frequency of KRAS mutations in prostate cancer patients from East Asian countries was much higher than that in American cases. In contrast to a very low prevalence in American patients (up to 3%) [25,31], about 7.3% of Korean patients [26], 9.1%–12.5% of Chinese patients [27,28], and 10%–17% of Japanese patients [25,29] harbor KRAS mutations. A similar difference in the frequency of BRAF mutation also has been reported in prostate cancer samples from Caucasian populations and men of Korean descent [26,33]. Furthermore, it has been recently described that although rearrangements of *BRAF* and *RAF1* occur at a comparable frequency between Chinese and Western samples, the prevalence of *BRAF* copy number gain in Chinese patients was significantly higher than that in patients for the United Kingdom (29% vs. 9.2%) [28]. Taken together, these results indicate that the RAS-RAF-MAPK signaling pathway may be more important for prostate cancer pathogenesis in Asian men than in Western men.

## 3 Castration-resistant prostate cancer and neuroendocrine differentiation

Despite the considerable differences in epidemiology and etiology of prostate cancer between Asian and Western countries, prostate cancer patients of different ethnic groups are currently treated with the same modalities mainly based on tumor grade and stage. We know very well now that a large portion of these patients have indolent tumors that will impact neither quality of life nor life expectancy, so that active surveillance is a preferred option. Localized cancers are treated with surgery or radiation with similar efficacy. For advanced and metastatic prostate cancer, hormonal therapy that inhibits androgen production and/or blocks androgen receptor (AR) function is the first-line treatment. However, many patients experience only a short term disease regression, and nearly all of them will eventually recur with castration-resistant prostate cancer (CRPC). During the past several decades, the cellular and molecular basis underlying the development of CRPC has been intensively investigated. Diverse mechanisms have been proposed, including sustained intratumoral synthesis of androgen, amplification of *AR* gene, gain-of-function mutations and alternative splice variants of AR, changes in co-regulatory molecules, ligand-independent activation of AR signaling as well as other AR-independent pathways that facilitate cancer cell survival and growth under androgen-depleted conditions [34]. Recently, an integrated genetic study of 50 heavily pre-treated CRPC samples obtained at rapid autopsy revealed the mutational landscape of lethal metastatic CRPC. In addition to previously reported recurrent genomic alterations such as *PTEN, AR, RB1, TP53* and *APC* mutations, several novel somatic mutations in multiple chromatin/ histone modifiers including MLL2, UTX and ASXL1 as well as transcription factors FOXA1 and ETS2 were found in CRPC [35].

In addition to these identified molecular alterations, a pathological characteristic referred as neuroendocrine differentiation of prostate cancer has also been demonstrated to be significantly associated with the development of CRPC [36–38]. Epithelia of mouse and human normal prostate consist of three types of differentiated cells: basal cells, luminal secretory cells, and neuroendocrine cells, which are proposed to be derived from a common pool of prostate stem/progenitor cells [39]. The luminal cells are columnar epithelial cells constituting the bulk of the polarized glandular structures, with high levels of AR expression and AR-dependent secretory machinery; whereas basal cells are localized between the luminal cells and the underlying basement membrane, and express p63 and relatively low levels of AR. Neuroendocrine cells constitute a minor population (~1%) of the total epithelial cells in the normal prostate, and are scattered throughout the epithelial compartment and can be detected by immunohistochemical (IHC) staining with neuroendocrine cell-specific markers such as chromogranin A or synaptophysin [38]. Due to the lack of nuclear AR expression in neuroendocrine cells [40,41], neuroendocrine cells in normal prostate are resistant to castration, while androgen ablation leads to apoptosis of the majority of luminal cells and growth arrest of basal cells [42].

**Figure 1:**
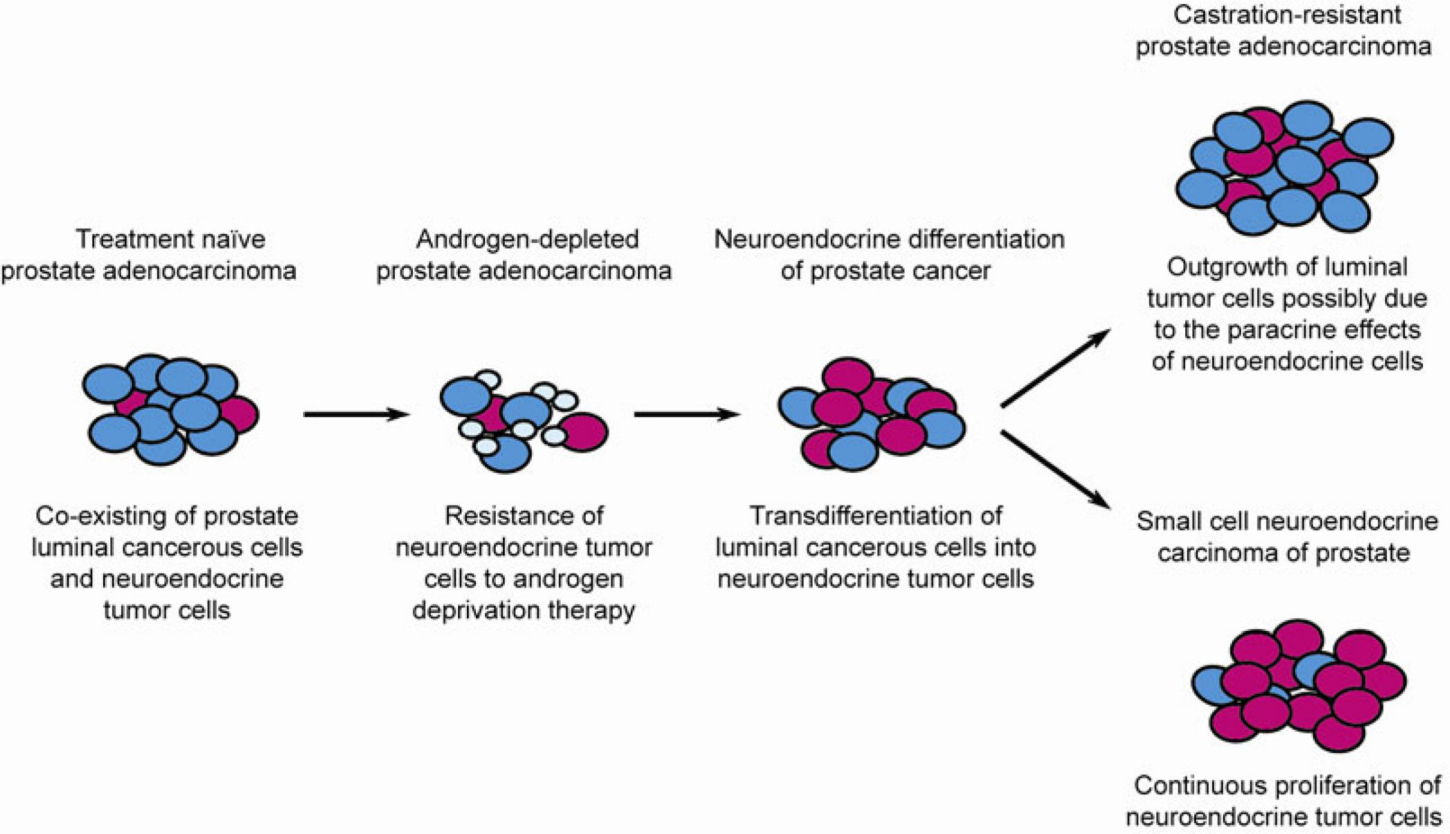
The model of neuroendocrine differentation of prostate cancer and the development of small cell neuroendocrine carcinoma after androgen deprivation therapy.

In prostate adenocarcinoma, the predominant histological subtype of human prostate cancer, the neoplastic glands are mainly composed of proliferating luminal type cancerous cells, and complete loss of basal cells. However, scattered or nests of neuroendocrine cells are also present in almost all cases of prostate adenocarcinoma [43], with varied ratios of neuroendocrine to acinar-type cells in different patients. Increasing evidence supports the notion that these neuroendocrine tumor cells are different from their counterparts in the normal prostate gland, in terms of their cellular morphology and expression of lineage/tumor-specific markers including cytokeratin 5/18 and AMACR [36,41], indicating that neuroendocrine tumor cells are one type of bona fide epithelial constituent of prostate cancer (Figure 1). Recent studies to assess *ERG* gene fusion status in neuroendocrine tumor cells by FISH analysis consistently showed that despite the lack of ERG protein expression, the *TMPRSS2-ERG* fusion can be detected in neuroendocrine cells that intermingle with *ERG* rearrangement positive adenocarcinoma component within the same tumor foci [44]. These findings further support the notion that unlike normal neuroendocrine cells, neuroendocrine tumor cells could share a common cellular origin with luminal type cancer cells.

Interestingly, neuroendocrine tumor cells also display several unique features distinct from luminal secretory-type cancer cells in prostate cancer. While highly proliferating luminal cancer cells are generally positive for AR and PSA, neuroendocrine cells in prostate adenocarcinoma are usually quiescent and lack expression of AR and PSA [40,41]. Due to these intrinsic features of neuroendocrine tumor cells, hormonal therapy of prostate adenocarcinoma usually causes an increase in neuroendocrine differentiation and sometimes induces the development of secondary small cell neuroendocrine carcinoma (SCNC) (Figure 1). Unlike adenocarcinoma, SCNC of the prostate does not show glandular structure but has a solid sheet-like growth pattern. The tumor cells are small with scant cytoplasm, finely granular and homogeneous chromatin and no prominent nucleoli. Nuclear molding, crush artifact, mitotic and apoptotic figures are common histologic findings. SCNC is extremely aggressive and is often widely metastatic at the time of diagnosis. It does not respond to hormonal therapy and usually leads to death within a year [37,38,45].

It has been increasingly recognized that neuroendocrine differentiation following hormonal therapy is associated with tumor progression and castration resistance [41,45,46]. However, it remains controversial whether there is a causal relationship between the increased neuroendocrine differentiation and CRPC development. Although the physiological roles of neuroendocrine cells in prostate organogenesis and functional regulation are largely unknown, it is proposed that neuroendocrine cells could interact with other types of prostate epithelial cells and stromal cells via various mechanisms, due to their dual properties of neurons and endocrine cells, such as dendrite-like cytoplasmic extensions and abundant neurosecretory granules containing histamine, serotonin, neuron-specific enolase and many other peptides/neuropeptides and cytokines [36]. In prostate cancer, especially under the androgen-deprived environment, neuroendocrine tumor cells may promote the androgen-independent growth of the luminal type prostate cancer cells [36]. Mechanistically, the growth-promoting function of neuroendocrine tumor cells may be mediated by the paracrine effects of peptide hormones such as bombesin/gastrin-releasing peptide family of neuropeptides, which are secreted from neuroendocrine tumor cells and could stimulate androgen-independent survival, growth and metastasis of the neighboring luminal type prostate cancer cells [47] (Figure 1).

## 4 The cellular origins and key genetic events for SCNC

To date, the cellular origin of neuroendocrine tumor cells in prostate adenocarcinoma is unclear. Given the pluripotency of prostate stem cells/progenitors that can give rise to basal, luminal and neuroendocrine cells in the regeneration assay [48–50], it is proposed that neuroendocrine tumor cells in prostate adenocarcinoma may be derived from prostate stem cells that are transformed by a series of oncogenic events. On the other hand, several preclinical studies showed that in an androgen-depleted setting or upon treatment with IL-6, EGF or other agents that elevate the intracellular cyclic AMP, luminal type cancer cells could undergo a process of transdifferentiation to acquire the morphology and lineage specific markers of neuroendocrine cells [51–55], suggesting that neuroendocrine tumor cells could be alternatively derived from luminal cancer cells in response to the pressure of surviving in an androgen-depleted condition. This transdifferentiation model of neuroendocrine tumor cells is further supported by a recent study of secondary SCNC, showing that prostate luminal cell lines (RWPE-1 and LNCaP) have the ability to transdifferentiate into cells with a neuroendocrine-like phenotype when they were stably transfected with transcription factor N-myc or Aurora kinase A (AURKA), a serine/theronine kinase involved in cell cycle regulation [44].

Using next-generation RNA sequencing and oligonucleotide arrays, Beltran et al. [44] showed that in contrast to the lower prevalence (5%) in localized prostate adenocarcinoma, about 40% of metastatic SCNC displayed concurrent overexpression and amplification of *AURKA* and N-myc gene (*MYCN*), indicating that N-myc and AURKA could play important roles in the development of SCNC of the prostate. Moreover, the preclinical studies with an AURKA inhibitor PHA-739358 demonstrated that this kinase antagonist has specific inhibitory effects on the growth of neuroendocrine tumor cells in cell culture and xenograft models [44], suggesting that enhanced AURKA kinase activity could be essential for the maintenance of SCNC. However, the definitive mechanisms underlying AURKA/N-myc-associated neuroendocrine differentiation remain unknown. The contributions of other genetic alterations, such as decreased expression of transcription factor REST and upregulation of epithelial-mesenchymal transition associated molecules [56], to neuroendocrine differentiation and tumor growth should be determined in future studies.

In contrast to the absence of proliferative activity of neuroendocrine cells in benign prostate tissues and prostate adenocarcinomas, neuroendocrine cells in primary (de novo) or secondary SCNC are highly proliferative, which results in almost all patients dying within one year following diagnosis [38,57,58]. The molecular mechanisms underlying the difference in the cell cycle status of neuroendocrine cells in benign prostate/adenocarcinoma and SCNC remain unclear. Our previous study demonstrated that interleukin-8 (IL-8), a cytokine potentially involved in androgen-independent growth of prostate cancer [59], and its receptor CXCR2 are exclusively expressed by neuroendocrine tumor cells in prostate adenocarcinoma [60]. Given the recent findings showing activation of CXCR2 by IL-8 leads to cellular senescence in a p53-dependent manner [61], we propose that the IL-8-CXCR2-p53 axis could be the major regulatory signaling pathway to maintain the neuroendocrine cells of benign prostate and adenocarcinoma in a quiescent state. In a recent study, we showed that the expression of wild-type p53 protein is required for the growth-inhibitory effects of IL-8-CXCR2 signaling on two different prostate cancer cell lines [62], which provides experimental evidence to support our hypothesis. Importantly, while neuroendocrine cells in benign prostate and adenocarcinoma express wild-type p53, IHC analysis of SCNC samples revealed that the majority of the NE tumor cells in SCNC display strong and diffuse nuclear p53 staining, suggesting that p53 is frequently mutated in SCNC [62]. Furthermore, targeted sequencing of exons 5–10 of *TP53* gene showed that five of seven cases of SCNC harbor a recurrent p53 mutation (D184N) [62], supporting the notion that p53 missense mutation in neuroendocrine cells could be the critical genetic event in the development of prostate SCNC.

## 5 Animal models of SCNC

The identification of p53 protein as a key molecular determinant for the regulation of neuroendocrine cell proliferation and quiescence not only offers the critical link between the transformation of neuroendocrine cells and the development of prostate SCNC, but also provides a mechanistic explanation for the neuroendocrine phenotype of prostate tumors from the transgenic adenocarcinoma of mouse prostate (TRAMP) model. The TRAMP transgenic mouse strain is engineered to express the SV40 virus large T and small t tumor antigens in prostate epithelial cells under the control of the AR-responsive rat *probasin* promoter [63,64]. These mice ultimately develop predominantly SCNC and metastasis to distant sites, with limited signs of the formation of prostate adenocarcinoma in some strains [65]. It is well accepted that SV40 T antigen-induced transformation is mainly mediated via inactivation of p53 and retinoblastoma (Rb) pathways [66], indicating that loss of these two tumor suppressors may substantially contribute to the development of SCNC. Consistent with the TRAMP model, a similar phenotype of prostate cancer with neuroendocrine differentiation was also observed in the compound knockout mice with prostate-specific deficiency in p53 and Rb, although either p53 deletion or Rb loss alone only resulted in prostatic intraepithelial neoplasia (PIN) in aged mice [67]. These data suggest that inactivation of both p53 and Rb pathways are required to cause the formation of SCNC in the mice. Our studies have demonstrated that p53 mutation is likely a critical molecular event for human SCNC but it remains to be determined if inactivation of Rb is also required.

In addition to genetically engineered mouse models, the tissue recombination/transplantation model of prostate cancer is a very efficient approach to rapidly interrogate the functional consequences of various genetic alterations central to the initiation and progression of the human disease [68]. By appropriate reconstitution of the epithelial-stroma interactions to mimic the native tumor microenvironment, the tissue recombination model consisting of adult prostate epithelial cells and embryonic urogenital sinus mesenchymal cells can faithfully recapitulate human prostate cancer evolution as well as the neoplastic lesions developed in transgenic mice [69,70]. Furthermore, by incorporating other genetic tools such as lentivirus-based gene transfer and shRNA-mediated knockdown, distinct subpopulations of epithelial cells and mesenchymal cells can be genetically manipulated with a high degree of flexibility. In a fast and cost-effective manner, this powerful system can test both the cell-autonomous roles of genetic events in the epithelial compartment and the influence of microenvironment on prostate carcinogenesis through paracrine actions [71–73].

Importantly, primary prostate epithelial cells freshly isolated from benign human prostate can be used as the starting material to study prostate cancer development in the tissue recombination model, which makes it possible to examine many fundamental differences in prostate cancer biology between mice and humans. Recently, we successfully established an *in vivo* transformation assay using naïve human prostate epithelial cells directly harvested from patients [74,75]. By coupling a lentiviral transduction technique with the *in vivo* regeneration approach, we demonstrated that CD49f^hi^Trop2^hi^ basal cells from primary benign human prostate tissue are able to initiate prostate cancer in immunodeficient mice. The synergistic effects of AKT activation, overexpression of ERG and AR in basal cells closely recapitulated the histological and molecular features of human prostate cancer, with loss of basal cells and expansion of malignant luminal cells expressing PSA and AMACR [74]. These findings suggest that basal epithelial cells can be a cell of origin for human prostate cancer. Although neuroendocrine differentiation is not observed in prostate tumors derived from lentiviral transduced human basal cells in our current tissue recombination model, further investigation of the cellular origins of SCNC and identification of genetic and epigenetic changes in neuroendocrine differentiation will facilitate the establishment of relevant mouse models of SCNC, providing insights into the pathogenesis of SCNC and the development of effective therapeutic strategies for this aggressive prostate malignancy.

## 6 Future direction

With continuous improvement in technologies and the significant reduction in cost for high-throughput sequencing, remarkable advances have been made in molecular characterization of prostate cancer. These findings at the molecular level are being translated into valuable diagnostic biomarkers and prognostic predictors in the clinic, offering an opportunity for patient stratification and personalized therapy. However, several unique features of prostate cancer, including vastly different biology of different tumors, tumor multifocality, tumor heterogeneity and the preference of bone as metastatic sites, pose significant challenges for the acquisition and analyses of tumor samples. The exact roles of many newly identified genetic alterations in prostate carcinogenesis remain uncertain. In addition, the contribution of other factors, such as epigenetic changes, to ethnic variation and histological subtype differences in prostate cancer has not been extensively investigated. Despite these limitations and challenges, the recent molecular findings have provided strong candidates for the development of novel targeted therapeutic agents. Combination therapies targeting multiple molecules and pathways will likely substantially improve the clinical outcome of patients with advanced prostate cancer in the near future.

## Figures and Tables

**Table 1:** The prevalence of key genetic events for prostate carcinogenesis among different ethnic groups

Genetic alteration	Prevalance in Asian patients (%)	Frequency in patients from Western countries (%)
*ERG-TMPRSS2* fusion	8–21 [19,21–23]	50–70 [17]
PTEN inactivation	34 [24]	70 [24,30]
*CTAGE-KHDRBS3* fusion	37 [21]	Unkown
*USP9Y-TTTY15* fusion	35 [21]	Unkown
KRAS mutations	7–17 [25–29]	Up to 3 [25,31]
*BRAF* copy number gain	29 [28]	9 [28]
